# MDA5 gain-of-function associated with a Glu794del mutation

**DOI:** 10.1007/s10875-024-01813-7

**Published:** 2024-10-02

**Authors:** Callie Wong, Lukas Gerasimavicius, Yanick J. Crow, Carolina Uggenti, E794del consortium, Jenny Carmichael, Hayley Lees, Gillian I Rice, Fraser JH Sutherland, Joseph A Marsh

**Affiliations:** 1 MRC Human Genetics Unit, Institute of Genetics and Cancer, University of Edinburgh, Edinburgh, UK; 2 Laboratory of Neurogenetics and Neuroinflammation, Institute Imagine, Paris, France

**Keywords:** IFIH1, MDA5, Interferonopathy, Deletion mutation, Gain-of-function

To the Editor

*IFIH1* encodes the melanoma differentiation associated gene 5 protein (MDA5) which senses viral double-stranded (ds) RNA in the cytosol, leading to the induction of a type I interferon mediated anti-viral response. Beginning in 2014, heterozygous gain-of-function (GOF) mutations in *IFIH1* have been described to cause a spectrum of systemic and neuroinflammatory phenotypes including classical Aicardi-Goutières syndrome (AGS) and Singleton-Merten syndrome. In 2020, we published a description of 74 individuals from 51 families segregating a total of 27 putative pathogenic mutations in MDA5 [1]. Notably, these, and all other such mutations reported to date, involved missense substitutions, considered likely to confer a gain of function through tighter dsRNA binding and subsequent increased baseline and ligand-induced interferon signalling. In contrast to all other previously described GOF mutations in MDA5, we now report a novel Glu794del mutation to cause characteristic features of AGS. A survey of the literature indicates that deletions conferring gain of function are extremely rare, but are important to recognise from a clinical perspective and can provide novel insights into molecular pathology.

We ascertained a female child (AGS3669) born to non-consanguineous parents of white European (British) ancestry with no significant family history of note. She was delivered vaginally after a normal pregnancy at 37 + 4 weeks gestation, weighing 2.49 kg. There were no immediate post-natal problems, and she was home on day 3 of life. Her development was normal until at least the age of 8 months, at which time she was sitting independently, weight-bearing, pulling herself to stand and babbling with meaning. At this age she developed a viral illness while on holiday, leading to attendance at an emergency department with a fever of 40 degrees where she was diagnosed with SARS-CoV-2 infection. After 4–5 days she had made a complete recovery. At 10 months of age she became ill with RSV bronchiolitis but did not require admission to hospital. Following on from this episode, it was noticed that she was “becoming vacant” and was “not herself”. She was seen urgently 2 weeks later because of a significant reduction of truncal tone and a loss of motor skills – so that she was no longer sitting independently or standing up, no longer vocalizing (apart from crying) or smiling, and had developed difficulties with swallowing. There was a rapid progression of these features, so that within 2 months she was extremely hypotonic, unaware of her surroundings, had lost all facial expression and required tube feeding. MRI of the head and spine at the age of 1 year was reported as normal. However, bilateral calcification of the basal ganglia, and at the grey-white matter junction of both cerebral hemispheres, was seen on cerebral computed tomography at the age of 22 months (Fig. 1A). Now, aged 2 years she demonstrates minimal truncal tone and head control, marked spasticity in the upper and lower limbs and some dystonic movements. There is no meaningful smiling or vocalisation and she is gastrostomy fed.

**Fig. 1:**
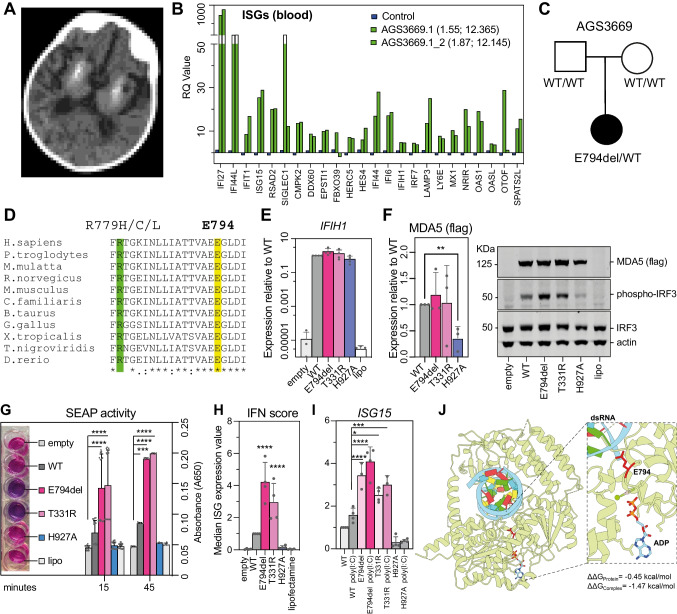
**A** Low resolution cerebral CT imaging at age 22 months demonstrating dense calcification of the basal ganglia and at the grey-white matter junction bilaterally. **B** Interferon stimulated gene (ISG) mRNA expression in peripheral whole blood assessed using NanoString technology. The first number in brackets is the decimalized age at the time of sampling, and the second the interferon score (calculated according to the median fold change in relative quantification of the ISGs compared with 27 controls (blue bars; normal < 2.75)). **C **Simplified pedigree structure and mutation status (WT = wild-type). Circles and square indicate females and male, respectively. Filled shapes indicate affected status. **D** CLUSTAL Omega alignment of MDA5 homologues. The glutamine deleted by the NM_022168.4:c.2381_2383del mutation in *IFIH1* is highlighted in yellow, and the recurrently mutated residue at position 779 in green. **E** IFIH1 expression (qPCR, n = 4). **F** MDA5 protein expression (WB, n = 3). **G** Colour change in Quanti-Blue solution (Left). SEAP activity (Right). **H** Composite interferon (IFN) score in HEK-Blue ISG cells transduced with the E794del mutant construct or the T331R or H927A constructs serving as positive and negative controls respectively (*n* = 4). **I** Expression of ISG15 before and after stimulation of HEK-Blue ISG cells with the dsRNA mimic poly(I:C), (*n* = 4). **J** The structural effects of the MDA5 E794 deletion were modelled in Rosetta, and their energetic impacts evaluated using the Relax application. The difference in Gibb’s free energy change for the complex (ΔΔG_Complex_) proved to be the most impacted by this deletion, demonstrating a lower ΔΔG value, stabilizing the protein interactions with dsRNA and the ADP ligand. PDB structure 7JL0 was used for the E794 visualization and deletion modelling. One way ANOVA with Dunnett’s multiple comparisons test **** = *p* < 0.0001, *** = *p* < 0.001, ** = *p* < 0.01, * = *p* < 0.05, ns = non specific

Assessment of interferon stimulated gene (ISG) expression in whole blood revealed scores of 12.365 and 12.145 at the ages of 1.55 and 1.87 decimalised years respectively (normal < 2.758) (Fig. 1B). At age 19 months, cerebrospinal fluid neopterin was elevated to 10 times, and tetrahydrobiopterin 3 times, the upper limit of normal.

Gene-agnostic trio genome analysis identified a heterozygous de novo three base-pair deletion of *IFIH1* (NM_022168.4:c.2381_2383del), leading to the loss of a single amino acid within the conserved core helicase domain of MDA5 (p.(Glu794del)(E794del)) (Fig. 1C). This in-frame deletion is not present on more than 1.4 million alleles recorded on gnomAD v4.1.0, and involves the removal of a highly conserved glutamate residue close to the recurrently mutated arginine at position 779 of MDA5 (Fig. 1D). Sequencing of the eight other genes known to be associated with AGS identified no pathogenic variants.

Using a E794del MDA5 plasmid, we observed RNA (Fig. 1E) and protein (Fig. 1F) expression at equivalent levels to wild-type (WT), and to a T331R variant previously seen in a patient with features of a type I interferonopathy and which was shown to confer a gain of function in vitro. In contrast, the expression of a H927A loss-of-function (LOF) mutant that disrupts RNA binding was significantly reduced compared to WT, consistent with this substitution conferring reduced protein stability.

A commercial SEAP (secreted alkaline phosphatase) reporter system was used to explore the effect of the E794del on interferon signalling. When interferon binds to the interferon receptor, the transcription of ISGs and SEAP is triggered simultaneously, and a read-out of SEAP secretion is used to determine interferon production with QuantiTM-Blue solution. Compared to WT MDA5, we observed a significantly higher SEAP reporter activity with both the E794del and the known T331R GOF mutation (Fig. 1G). Of note, a slight increase in SEAP activity was recorded with the WT construct at 45 min compared to the empty and lipofectamine-only controls, likely because of competitive binding due to overexpression of MDA5.

To validate the results of the SEAP reporter assay, RT-qPCR was conducted to examine the expression of six selected ISGs. Results were combined to produce the interferon (IFN) score, calculated as the median value of the expression level of the six ISGs tested. An increased interferon score was seen with the E794del and T331R mutant constructs (Fig. 1H). Further, the phosphorylation of IRF3, a key molecule for the initiation of the interferon response, was observed in cells transfected with E794del and T331R (Fig. 1F). Upon stimulation with poly(I:C), a double-stranded homopolymer used to model dsRNA, we did not observe further activation of the E794del and T331R mutant proteins, suggesting that the E794del MDA5 mutant capacity to bind RNA is saturated at steady state (Fig. 1I).

MDA5 complex structures from the protein data bank (PDB) containing relevant ligands (dsRNA, ADP) were used to generate E794del mutant structures, and PDB structure 7JL0 was selected for further analyses. Remodelling was carried out for the deletion and WT under two scenarios – protein only, or in full complex with the viral dsRNA ligands available in 7JL0. An assessment of the difference in Gibb’s free energy change for the complex (ΔΔG_Complex_) suggested a ΔΔG of -1.47 kcal/mol for the MDA5 complex with dsRNA and ADP, supportive of an overall increase in stability (Fig. 1J). Of note, the ΔΔG of the deletion was -0.45 kcal/mol when considering the monomeric protein itself, suggesting that most of the overall stabilisation is due to the intermolecular interactions between the mutant protein, dsRNA and ADP. Analogously, the effects of the T331R point substitution were evaluated using a protocol developed for amino acid substitutions. This mutation was predicted to be destabilising, but with a considerably milder effect observed for the full complex (1.14 kcal/mol) compared to the monomer structure alone (3.81 kcal/mol). The mutation being less destabilising in the complex could imply that any unfavourable energetic effects of the variant are offset by a stronger binding of the dsRNA and/or ATP, in line with a GOF mechanism.

MDA5 is a member of the retinoic acid inducible gene-I (RIG-I) receptor family. Recognition of cytoplasmic viral dsRNA by MDA5 induces filament assembly along the dsRNA axis, with the helicase domains and C terminal domain (CTD) responsible for RNA recognition. Filament formation then results in oligomerization of the tandem CARD domains (2CARD) of MDA5, leading to the interaction with mitochondrial MAVS, and subsequent induction of interferon and other pro-inflammatory cytokines. MDA5 filament stability is intrinsically regulated by ATP hydrolysis, which is stimulated upon dsRNA binding.

While premature termination mutations in the helicase domain of MDA5 are seen in control populations as common polymorphisms, LOF mutations in MDA5 have been reported to underlie recurrent viral infections of the upper and lower respiratory tract, as well as enterovirus encephalitis. In contrast, GOF mutations result in a spectrum of systemic and neuroinflammatory phenotypes. Notably, all ascertained type I interferonopathy-associated mutations described to date have been missense variants, likely conferring increased sensitivity to self-derived dsRNA. The clustering of mutations near the ATP binding region highlights increased RNA binding affinity, and / or decreased efficiency of ATP hydrolysis and filament disassembly, as fundamental to the molecular pathology of these substitutions. We hypothesize that deletion of E794 could be stabilising to the protein-dsRNA complex by removing the negatively charged glutamate side chain, which is in close proximity to the phosphate backbone in the WT structure.

Although the diversity of molecular mechanisms associated with pathogenic missense variants has been studied systematically, little is known about how other types of coding variation (e.g. in-frame deletions or insertions of varying size) are associated with LOF *vs* GOF. Examples of deletions conferring a gain of function at a molecular level are apparently very rare. While biallelic LOF, or heterozygous dominant negative, mutations in interleukin-6 signal transducer (IL6ST), encoding the GP130 protein (which functions in the transduction of IL6 cytokine family signalling), cause hyper-IgE recurrent infection syndrome, Materna-Kiryluk et al. [2] described a de novo, mosaic, Tyr186_Tyr190del in GP130 to confer constitutive GP130 cytokine receptor signalling. Further, several similar, somatic, deletions have been described in hepatocellular tumours, all of which are predicted to disrupt key residues involved in the GP130–IL-6 interface, with mutant protein capable of homodimerization, or heterodimerization with WT gp130, independently of IL-6 [3]. As another example, CARD11 is a large multi-domain scaffold protein uniquely expressed in the haemopoietic lineage, responsible for coordinating signalling events downstream of the B / T cell receptor. Germline CARD11 GOF mutations cause B cell expansion with NF-κB and T cell anergy (BENTA) disease. In contrast, heterozygous LOF dominant negative mutations are causative of CARD11-associated atopy with dominant interference of NF-kB signalling (CADINS) [4]. Notably, while most CARD11 GOF mutations are missense, single amino acid deletions (K215del: [5]; G123D: [6]) have been shown to confer a gain of function, likely reflective of uncharacterised effects on the signalling complex of which CARD11 is a component. Finally, to our knowledge, single amino acid deletions conferring gain of function have also been reported once in STAT1 (L301del: [7]) and STAT3 (E616del: [8]), among, respectively, more than 100 and 25 such mutations all of which are otherwise missense (although with no proven in vitro evidence in the case of the L301del in STAT1).

## Supplementary Information

Below is the link to the electronic supplementary material.

ESM 1 (DOCX 20.9KB)

## Data Availability

No datasets were generated or analysed during the current study.

## References

[1] Rice GI, et al. Genetic and phenotypic spectrum associated with IFIH1 gain-of-function. Hum Mutat. 2020;41:837–49. 31898846 10.1002/humu.23975 PMC7457149

[2] Materna-Kiryluk A, et al. Mosaic IL6ST variant inducing constitutive GP130 cytokine receptor signaling as a cause of neonatal onset immunodeficiency with autoinflammation and dysmorphy. Hum Mol Genet. 2021;30:226–33. 33517393 10.1093/hmg/ddab035

[3] Rebouissou S, et al. Frequent in-frame somatic deletions activate gp130 in inflammatory hepatocellular tumours. Nature. 2009;457:200–4. 19020503 10.1038/nature07475 PMC2695248

[4] Ma CA, et al. Germline hypomorphic CARD11 mutations in severe atopic disease. Nat Genet. 2017;49:1192–201. 28628108 10.1038/ng.3898 PMC5664152

[5] Shields AM, Bauman BM, Hargreaves CE, Pollard AJ, Snow AL, Patel SY. A Novel, Heterozygous Three Base-Pair Deletion in CARD11 Results in B Cell Expansion with NF-κB and T Cell Anergy Disease. J Clin Immunol. 2020;40:406–11. 31897776 10.1007/s10875-019-00729-x

[6] Takase Y, et al. A familial case of B-cell expansion with NF-κB and T-cell anergy caused by a G123D heterozygous missense mutation in the CARD11 gene. Pediatr Blood Cancer. 2022;69: e29941. 36129242 10.1002/pbc.29941

[7] Toubiana J et al.; International STAT1 Gain-of-Function Study Group. Heterozygous STAT1 gain-of-function mutations underlie an unexpectedly broad clinical phenotype. Blood 2016;127:3154–3164. 10.1182/blood-2015-11-679902 PMC4920021 27114460

[8] Gutiérrez M, et al. Partial growth hormone insensitivity and dysregulatory immune disease associated with de novo germline activating STAT3 mutations. Mol Cell Endocrinol. 2018;473:166–77. 29378236 10.1016/j.mce.2018.01.016 PMC6143347

