# Supplementary material for: MDA5 gain-of-function associated with a Glu794del mutation
Source: J Clin Immunol. 2024 Oct 2;45(1):20. doi: 10.1007/s10875-024-01813-7 (PMC11447049; doi:10.1007/s10875-024-01813-7)
Supplement: Supplementary file 1 — (DOCX 20.9KB) [file 10875_2024_1813_MOESM1_ESM.docx]

**MDA5 gain-of-function associated with a Glu794del mutation**

Callie Wong^1^, Lukas Gerasimavicius^1^, E794del Consortium, Yanick J Crow,^1,2^ Carolina Uggenti^1^

^1^MRC Human Genetics Unit, Institute of Genetics and Cancer, University of Edinburgh, Edinburgh, United Kingdom

^2^Laboratory of Neurogenetics and Neuroinflammation, Institute Imagine, Paris, France

**Supplementary information**

**Methods**

**Genetic analysis**

Rapid diagnostic trio genome analysis was undertaken with libraries prepared using Illumina DNA PCR-Free Prep according to the manufacturer’s instructions, and an Illumina NovaSeq 6000 (Illumina, San Diego, CA, USA). Variant calling / filtering was performed as previously described (1), based on the reference cDNA sequence NM_022168.2. Population allele frequencies were obtained from the gnomAD database (http://gnomad.broadinstitute.org). Evidence for variant pathogenicity was assessed according to ACMG/AMP and ACGS 2020 variant classification guidelines ((2); https://www.acgs.uk.com/media/11631/uk-practice-guidelines-for-variant-classification-v4-01-2020.pdf).

**In-vitro MDA5 expression**

Glu794del plasmid construction: The Q5 Site-Directed Mutagenesis (E0554) kit was used to construct the CMV4 plasmid with *IFIH1*, c.2381_2383del following the manufacturer’s instructions. *IFIH1*/MDA5 UniProtKB identifier: Q9BYX4. Plasmids were isolated with the MIDI KIT (Qiagen, 12143) and sequenced to select mutant clones. Cell Culture: HEKBlue^TM^ ISG cells were cultured with DMEM, GlutaMax + 10%FBS, 100ug/mL normocin, 100ug/mL zeocin (complete media). Splitting and subculture was undertaken twice per week. Cells were seeded onto six-well plates for later transfection and MDA5 overexpression. Opti-MEM GlutaMax and Lipofectamine2000 were used for delivery of 1ug of wild-type (WT), E794del, T331R (3) and H927A (4) MDA5 constructs, following the manufacturer’s instructions. Empty CMV4 plasmid and lipofectamine (lipo) were used as controls. Cells were incubated in Opti-MEM GlutaMax + 5% FBS and transfection media for a minimum of 6h incubation. Media was then replaced with cell culture media, and gene expression assessed by qPCR after 24h or cells were treated with poly(I:C).

Poly (I:C) stimulation: 0.5 ug/ul polyinosinic–polycytidylic acid (Poly(I) • Poly(C)) or poly (I:C) was transfected into the cells using Lipofectamine2000 according to the manufacturer’s instructions. Cells were incubated overnight and gene expression was assessed by qPCR.

**Type 1 interferon signalling**

***Western blot***: For total protein analysis, cells were lysed in radioimmunoprecipitation assay (RIPA) buffer for 30 min on ice (Thermo Fisher Scientific) supplemented with cOmplete™ ULTRA Tablets, Mini, EDTA-free, EASYpack Protease Inhibitor Cocktail (Roche), PhosSTOP™ Phosphatase Inhibitor (Roche) and 0.1 mM of PMSF solution (Sigma-Aldrich). Cell lysates were then incubated for 20 min at 37 °C with 1.25 U μl−1 Benzonase Nuclease (Sigma-Aldrich) to digest DNA, and samples centrifuged at 10,000 r.p.m. for 10 min. Supernatant containing soluble protein fraction was collected and western blot analysis was performed as described previously (5). Primary and secondary antibodies used in this study were: Rabbit anti-Flag: DYKDDDDK (14793s, CST), Rabbit anti-MX1 (37949s, CST), Mouse anti-b-actin (3700s, CST), Donkey anti-Mouse (925-68072, LI-COR), Goat anti-Mouse (925-33210, LI-COR), Goat anti-Rabbit (925-32211, LI-COR), and Goat anti-Rabbit (925-68071, LI-COR).

***QuantiTMBlue Assay***: Quanti-blue solution was prepared according to the manufacturer’s protocol. 50uL of cell media (supernatant) from HEKBlueTM ISG cells and 450uL QuantiTM-Blue solution were added to each well of a 24-well plate for absorbance measurement at 15- and 45-minute timepoints (650nm). RT-qPCR: Total RNA was extracted, and DNase treated using Direct-zol kit (Zymo, R2050). Reverse transcription was performed with High-Capacity cDNA Reverse Transcription Kit using random hexamers and 1μg of RNA (Applied Biosystems). Gene expression was assessed by qRT-PCR using the TaqMan gene expression assay (Thermo Fisher Scientific) and QuantiStudio 5 Real-Time PCR systems. The probes used in this study were: Hs00223420_m1, IFIH1; Hs01086370_m1, IFI27; Hs00192713_m1, ISG15; Hs00199115_m1, IFI44L; Hs00356631_g1, IFIT1; Hs02786624_g1, GAPDH; Hs00895608_m1, MX1; and Hs00973637_m1, OAS1.

**Protein modelling**

MDA5 complex structures from the PDB, containing relevant ligands (dsRNA, ADP), were used to generate E794del mutant structures using the Rosetta Remodel application. PDB structure 7JL0 appeared amenable to loop remodelling through kinematic closure (KIC) and was selected for further analyses after removing non-MDA5 proteins from the assembly. Mutant structures were generated by deleting the E794 residue and remodelling 4 residues in both directions of the chain. WT structures also underwent remodelling as a control, with the 9 residue stretch centred on E794 undergoing loop KIC. Remodelling was carried out for the deletion and WT under two scenarios – protein only, or in full complex with the ligands available in 7JL0. Loop refinement was carried out for all remodelled structures, yielding 100 refined models each. To further minimize the energetics of the structures and assess the impact of the deletion, each refined model was used in the Rosetta Relax application to produce 1,000 relaxed structures in each case. Deletion ΔΔG values were calculated as previously described (6), where the mutant structure energy is multiplied by a coefficient that takes into account the change in residue number, compared to the WT:

$$\Delta\Delta G=n\left( \frac{{\Delta G}_{del}}{n-1} \right)-{\Delta G}_{wt}$$

where *n* is the number of residues, nucleotides and small molecules in the structure. To calculate the complex and monomer ΔΔG values the Relax scores of 10 most stable mutant and WT structures were averaged in either case to produce ΔG.

The T331R substitution was evaluated using Rosetta through a different, previously published Cartesian ΔΔG protocol (7) The same PDB structure (7JL0) was used after removing non-MDA5 proteins and crystallographic artefact ligands. The monomeric and full complex structures were relaxed throughout 25 iterations and the lowest energy structures were used for variant evaluation. The mutation ΔΔG value represents the mean of a triplicate run.

In both cases, the intermolecular energetic contributions of the mutations were estimated by comparing the ΔΔG values resulting from the monomer and complex structures.

**Supplementary references**

1. Chen W et al. Clinical and molecular characterization of novel FARS2 variants causing neonatal mitochondrial disease. Mol Genet Metab 2023;140:107657.

2. Richards S et al. Standards and guidelines for the interpretation of sequence variants: a joint consensus recommendation of the American College of Medical Genetics and Genomics and the Association for Molecular Pathology. Genet Med 2015;17:405-24.

3. de Carvalho LM et al. Musculoskeletal disease in MDA5‐related type I interferonopathy: A Mendelian mimic of Jaccoud's arthropathy. Arthritis Rheumatol 2017;69:2081–2091.

4. Rice GI et al. Gain-of-function mutations in IFIH1 cause a spectrum of human disease phenotypes associated with upregulated type I interferon signaling. Nat Genet 2014;46:503-509.

5. Uggenti C et al. cGAS-mediated induction of type I interferon due to inborn errors of histone pre-mRNA processing. Nat Genet 2020;52:1364-1372.

6. Woods H et al. Computational modeling and prediction of deletion mutants. Structure 2023;31:713-723.e3.

7. Hahnbeom Park X et al. Simultaneous optimization of biomolecular energy function on features from small molecules and macromolecules. J Chem Theory Comput 2016;12:6201-6212.
